# Efficacy and safety of ceftazidime-avibactam versus tigecycline in the treatment of pneumonia caused by carbapenem-resistant *Klebsiella pneumoniae*

**DOI:** 10.3389/fmed.2026.1879917

**Published:** 2026-08-28

**Authors:** Tongfei Yang, Mao Xu, Lu Cao, Yaling Shi, Xianpeng Shi

**Affiliations:** 1 Department of Pharmacy, Shaanxi Provincial People’s Hospital, Xi'an, Shaanxi, China; 2 Department of Pharmacy, Hubei Cancer Hospital, Tongji Medical College, Huazhong University of Science and Technology, Wuhan, Hubei, China

**Keywords:** carbapenem-resistant *Klebsiella pneumoniae*, ceftazidime-avibactam, clinical efficacy, mortality risk factors, pulmonary infection, tigecycline

## Abstract

**Background:**

Carbapenem-resistant *Klebsiella pneumoniae* (CRKP) causes severe high-mortality nosocomial pneumonia. Ceftazidime-avibactam (CAZ-AVI) and tigecycline (TGC) are key CRKP antimicrobials, but direct comparative efficacy and safety data are limited. This retrospective study compared clinical efficacy and safety of CAZ-AVI-based versus TGC-based regimens for CRKP pneumonia, and explored factors associated with all-cause mortality to inform clinical practice.

**Methods:**

A total of 63 patients with CRKP pulmonary infection admitted to Shaanxi Provincial People's Hospital from January 2023 to May 2025 were retrospectively enrolled. Patients were divided into the CAZ-AVI-based regimen group (*n* = 33) and the TGC-based regimen group (*n* = 30) according to their treatment regimen. Differences in therapeutic efficacy, microbiological clearance, and adverse events between the two groups were compared, and logistic regression analysis was performed to explore factors associated with all-cause mortality.

**Results:**

No statistically significant differences in baseline clinical characteristics were observed between groups (*P* > 0.05). Bacterial clearance rate was significantly higher in the CAZ-AVI than TGC group (72.73% vs. 36.67%, *P* = 0.004). No statistically significant differences were seen in clinical response, 28-day mortality, overall in-hospital mortality, superinfection, or hepatic and renal adverse events (*P* > 0.05). However, coagulation abnormalities (70.00% vs. 27.27%, *P* < 0.001) and thrombocytopenia (43.33% vs. 15.15%, *P* = 0.013) were significantly higher in the TGC than CAZ-AVI group. Firth penalized multivariable logistic regression identified exploratory associations of advanced age (adjusted OR = 1.12, 95% CI 1.03–1.22, *P* = 0.011) and comorbid diabetes mellitus (adjusted OR = 6.24, 95% CI 1.23–31.57, *P* = 0.027) with increased all-cause mortality. These preliminary findings warrant caution; treatment regimen showed no significant association with all-cause mortality.

**Conclusions:**

In this exploratory retrospective cohort, CAZ-AVI-based regimens were associated with a higher bacterial clearance rate and a lower incidence of coagulation and platelet abnormalities, while no statistically significant differences were observed in clinical efficacy or survival outcomes between the two regimens. Advanced age and comorbid diabetes mellitus showed exploratory associations with increased all-cause mortality, which warrant attention in clinical practice.

## 1 Introduction

Carbapenem-resistant Enterobacterales (CRE) have emerged as an urgent public health threat, and represent one of the leading causes of treatment failure and mortality in patients with nosocomial infections globally. Among CRE pathogens, carbapenem-resistant *Klebsiella pneumoniae* (CRKP) is the most prevalent epidemic pathogen in clinical practice, with a continuously and rapidly increasing prevalence and high associated morbidity and mortality (1, 2). Consistent surveillance data have demonstrated the worsening antimicrobial resistance crisis of CRKP in recent years. Between 2015 and 2020, the detection rate of CRKP in China increased by more than 60%, with a sustained upward trend observed across various provinces. Notably, the detection rate of CRKP has consistently remained at a high level in patients with hospital-acquired pneumonia (HAP) and ventilator-associated pneumonia (VAP) (1). Patients with CRKP-induced pulmonary infection have an extremely poor clinical prognosis, with an attributable mortality rate exceeding 40%. This condition not only significantly prolongs hospital length of stay and increases medical burden, but also poses formidable challenges to infection prevention and control, as well as clinical management in intensive care units (ICUs) (3, 4). Against the backdrop of the high pathogenicity and mortality of CRKP infections, the armamentarium of effective therapeutic agents available for clinical use remains extremely limited. Over the past decade and more, only a handful of agents, exemplified by ceftazidime-avibactam, have been successfully introduced into clinical practice; however, resistant strains have emerged rapidly after their marketing, and the pace of antibiotic development lags far behind the evolutionary progression of bacterial resistance (5). While phage therapy has exhibited definitive bactericidal potential in *in vitro* investigations, no commercially approved therapeutic products are available worldwide, restricted by barriers such as the clonal heterogeneity of CRKP strains and the formidable difficulties in formulation standardization (6, 7). Vaccination represents a core strategy for long-term prevention and control, yet current in silico epitope design approaches suffer from low efficiency and fail to match the dynamic, site-specific antigen expression profiles of *Klebsiella pneumoniae* during infection, which accounts for the failure of most vaccine candidates to achieve clinical translation (8). Accordingly, optimizing anti-infective regimens based on currently available pharmaceuticals has emerged as both a prominent research focus and a persistent challenge in the global field of anti-infective research.

At present, ceftazidime-avibactam (CAZ-AVI) and tigecycline (TGC) have been established as the key therapeutic agents for the clinical management of CRKP pulmonary infection in China (9). CAZ-AVI irreversibly inhibits class A (*Klebsiella pneumoniae* carbapenemase, KPC), class C, and partial class D β-lactamases via its avibactam moiety, thereby restoring the antibacterial activity of ceftazidime. It exhibits an *in vitro* susceptibility rate of over 97% against CRKP strains producing KPC and OXA-48-like enzymes, and multiple studies have confirmed its excellent clinical cure rate and microbiological clearance rate in the treatment of CRKP pneumonia (10, 11). However, this agent is inherently inactive against strains producing metallo-β-lactamases (MBLs). Furthermore, acquired resistance caused by KPC gene mutations has been observed in clinical practice, which significantly limits its long-term application (12, 13). TGC maintains high susceptibility to the vast majority of CRKP strains and has favorable tissue penetration in lung tissue, making it a critical component of clinical combination treatment regimens (14, 15). Nevertheless, TGC has distinct pharmacokinetic limitations: at conventional doses, its plasma concentration is low, and drug exposure in the alveolar epithelial lining fluid (ELF) often fails to achieve the optimal pharmacodynamic (PD) target, resulting in a high clinical failure rate of monotherapy for severe CRKP pneumonia. Meanwhile, its adverse events, including gastrointestinal reactions, hepatic impairment, coagulation abnormalities, and thrombocytopenia, further restrict its widespread clinical application (16, 17). Against this background of stalled development in novel antibiotics, phage therapy and vaccines, CAZ-AVI and TGC remain the two most widely accessible last-line therapeutic options for CRKP pneumonia in routine clinical practice, with few alternative strategies expected to enter widespread clinical application in the short term.

Although CAZ-AVI and TGC have been widely used in the clinical treatment of CRKP pulmonary infection, large-scale randomized controlled trials (RCTs) that directly compare the therapeutic efficacy and safety of monotherapy or core regimens based on the two agents remain relatively scarce (18). Several studies have shown that CAZ-AVI-based regimens are significantly superior to TGC-based regimens in terms of clinical cure rate and microbiological clearance rate in patients with CRKP pneumonia, while no statistically significant difference in 28-day survival rate is observed between the two groups (16, 19). Existing real-world studies have mostly focused on ICU patient populations, and systematic analyses of the bacterial clearance efficiency, impact on coagulation system safety, and risk factors associated with all-cause mortality in Chinese patients with CRKP pulmonary infection treated with the two regimens are still very limited (3, 16, 20). Meanwhile, there are significant differences in the combination modes of the two agents in clinical practice, and their potential impact on treatment outcomes has not been fully elucidated. Therefore, more clinical data based on the native Chinese population are urgently needed to clarify the clinical value, applicable scenarios, and safety profiles of the two regimens, so as to provide an evidence-based foundation for clinical decision-making (12, 21).

Against this grim clinical background, we conducted a retrospective analysis of the clinical data from 63 patients with CRKP-induced pulmonary infection who were diagnosed at Shaanxi Provincial People's Hospital between January 2023 and May 2025. We systematically compared the differences between the two treatment strategies of CAZ-AVI-based and TGC-based treatment strategies in terms of clinical response rate, bacterial clearance rate, patient survival prognosis, and the incidence of adverse events, and further explored the independent risk factors for all-cause mortality in these patients using a logistic regression model. The primary aim of this study was to supplement single-center real-world data on the treatment of CRKP pulmonary infections in China, clarify the differences in clinical efficacy and safety profiles between these two core antimicrobial agents, and ultimately provide an evidence-based reference for the optimal selection of the limited last-line therapeutic regimens for CRKP pulmonary infection in clinical practice.

## 2 Methods

### 2.1 Study design and participants

**Figure 1:**
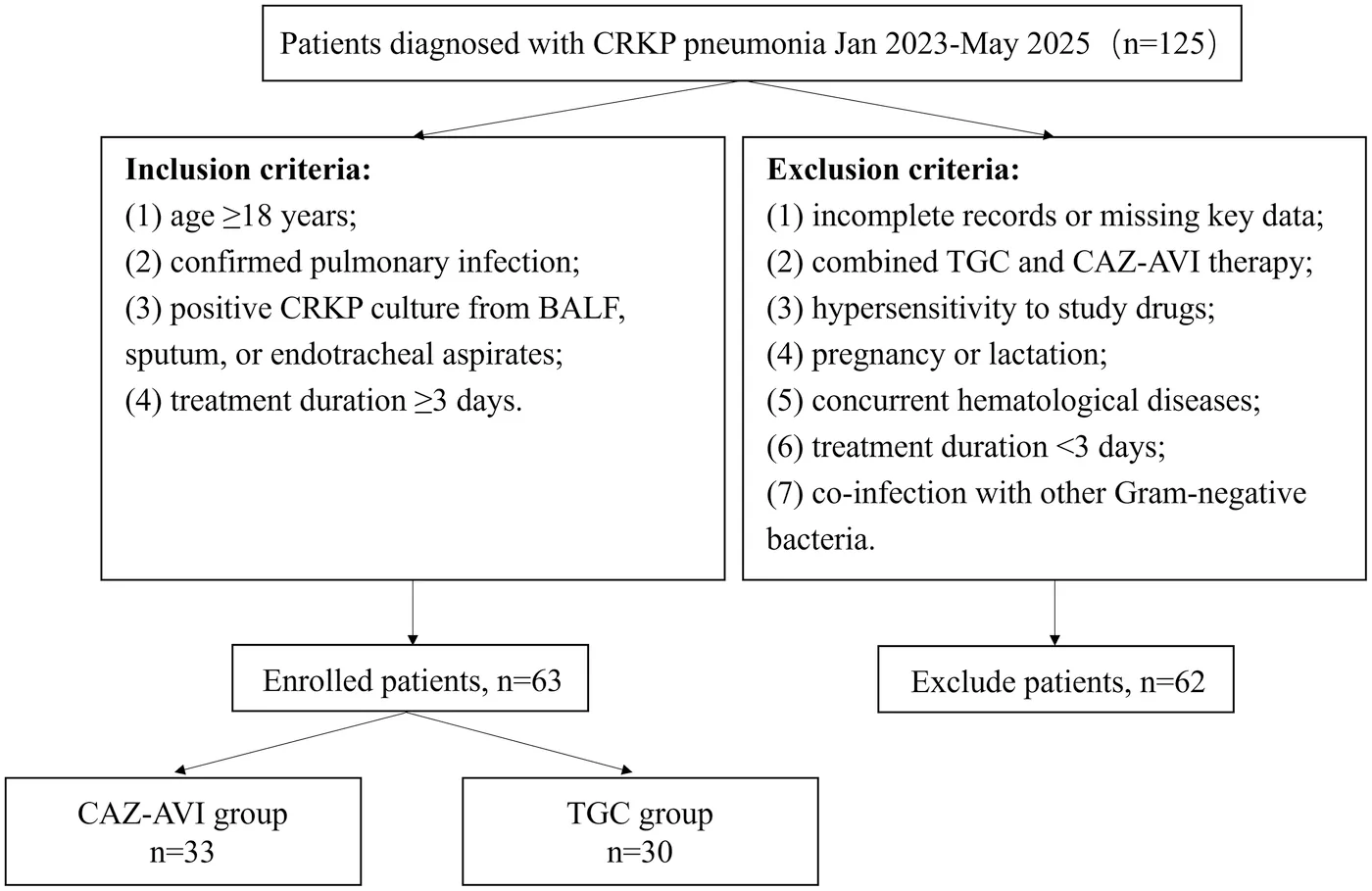
Study design.

This retrospective cohort study was conducted at Shaanxi Provincial People's Hospital, a tertiary teaching hospital in Northwest China. Consecutive patients admitted to both intensive care units and general wards with definitively diagnosed CRKP-induced pulmonary infection between January 2023 and May 2025 were retrospectively screened for eligibility. A total of 125 patients were initially identified; after the application of prespecified inclusion and exclusion criteria, 62 patients were excluded and 63 eligible subjects were finally enrolled, who were then divided into the CAZ-AVI group (*n* = 33) and the TGC group (*n* = 30) according to the core agent of the initial definitive CRKP-targeted antimicrobial regimen. The detailed patient enrollment and grouping process is illustrated in Figure 1.

The diagnosis of CRKP-associated hospital-acquired pneumonia or ventilator-associated pneumonia was established in accordance with the IDSA/ATS clinical practice guidelines (22). All enrolled patients met both clinical-radiological and microbiological criteria: presence of new or progressive pulmonary infiltrates on chest imaging plus at least two clinical signs of infection (body temperature > 38°C or < 36°C, peripheral white blood cell count > 10 × 10⁹/L or < 4 × 10⁹/L, or purulent respiratory secretions), together with positive culture of CRKP from qualified lower respiratory tract specimens including endotracheal aspirates, bronchoalveolar lavage fluid, or expectorated sputum; patients with only respiratory bacterial colonization, defined as positive culture without consistent clinical and radiological evidence of active infection, were excluded to ensure the homogeneity of the study population.

Inclusion criteria: (1) Aged ≥ 18 years old; (2) Clinically confirmed diagnosis of pulmonary infection; (3) CRKP infection verified by positive culture results of specimens including bronchoalveolar lavage fluid (BALF), qualified sputum specimens, or endotracheal aspirates; (4) Duration of the study treatment ≥ 3 days.

Exclusion criteria: (1) Incomplete medical records or missing key clinical data that precluded efficacy evaluation; (2) Patients who received a combination anti-infective regimen of TGC and CAZ-AVI; (3) Patients with hypersensitivity to either of the study drugs (TGC or CAZ-AVI); (4) Pregnant or lactating women; (5) Patients with concurrent hematological diseases; (6) Treatment duration < 3 days; (7) Patients with concurrent infection of other Gram-negative bacteria.

### 2.2 Microbiological testing

All lower respiratory tract specimens were processed and tested in compliance with the CLSI QMS18 quality management standard to ensure pre-analytical and analytical quality control (23). Bacterial identification was performed using the VITEK 2 Compact automated system. Antimicrobial susceptibility testing was conducted via the automated broth microdilution method, and minimum inhibitory concentration (MIC) results were interpreted according to the Clinical and Laboratory Standards Institute (CLSI) M100 breakpoints. Carbapenem resistance was defined as an MIC of imipenem or meropenem ≥ 4 mg/L. Escherichia coli ATCC 25922 was used as the quality control strain for all susceptibility assays.

### 2.3 Observation indicators

Baseline clinical characteristics of patients in the two groups were collected, including age, sex, body mass index (BMI), past medical history and personal history, total length of hospital stay, length of ICU stay, duration of mechanical ventilation, proportion of patients undergoing tracheotomy, concomitant infection status, treatment duration of the two study regimens, and detailed information on concomitant antibacterial medication (specific drug classes, daily dosages, duration of combination therapy, and *in vitro* susceptibility profiles of the isolates to these agents where available). The observation indicators for therapeutic efficacy included the clinical response rate, bacterial clearance rate, superinfection rate, and in-hospital mortality of the two groups. The safety observation indicators covered the incidence of treatment-emergent laboratory abnormalities in liver function, renal function, coagulation function, and platelet count during the treatment period. Of note, these abnormalities were observed events during treatment without formal causality assessment, and did not represent confirmed drug-related adverse reactions.

Clinical Response Rate: Clinical response was evaluated at the end of treatment, determined by comprehensive improvement or resolution of clinical symptoms, chest imaging findings, and serum inflammatory markers, together with microbiological eradication status. Clinical efficacy was classified as follows (16, 24): (1) Clinical response: Complete resolution of clinical symptoms and signs of infection, complete or partial eradication of the causative pathogen, and partial or complete normalization of non-microbiological indicators including imaging and laboratory examinations at the end of treatment. (2) Clinical failure: No significant improvement, partial or complete exacerbation of clinical symptoms and signs of infection, or development of new symptoms and signs related to the infection at the end of treatment.

Microbiological Clearance Rate: Microbiological clearance rate was assessed by comparing the microbial culture results before and after drug administration between the two groups, and the bacterial eradication status was determined accordingly. Microbiological efficacy was classified into the following categories (16, 25): (1) Eradication: No pathogenic bacteria causing the original infection were isolated from specimens collected from the primary infection site after treatment. (2) Presumed Eradication: Bacteriological results were defined as presumed eradication when sputum specimens were unobtainable due to complete resolution of clinical symptoms and signs, or when no specimens were submitted for culture after treatment. (3) Persistence: The original pathogenic bacteria of the infection were still isolated from specimens collected from the primary infection site after treatment. (4) Presumed Persistence: For patients judged as clinical failure, the pathogenic bacteria were presumed to be persistent when post-treatment culture was not performed or could not be performed. For the purpose of statistical analysis, eradication, presumed eradication and partial eradication were combined to calculate the overall clearance rate, while persistence and presumed persistence were combined to calculate the non-clearance rate.

Superinfection Rate: The superinfection rate after treatment was observed for patients in the two groups.

Mortality: The 28-day all-cause mortality was calculated from the date of initiation of the study antimicrobial regimen, and overall in-hospital mortality was also recorded for patients in the two groups. All patients were followed up until hospital discharge or death, with no loss to follow-up.

Safety Indicators: Safety outcomes were defined as new-onset laboratory abnormalities occurring from the first dose to the last dose of the study treatment. (1) Hepatic function abnormality was defined as an increase in alanine aminotransferase (ALT) of more than 1-fold the upper limit of normal (ULN) after drug administration, or an increase in total bilirubin (TBIL) of more than 1-fold the ULN after drug administration in patients without underlying liver disease (26). (2) Renal function abnormality was defined as an increase in serum creatinine (SCr) of more than 1.5-fold the ULN after drug administration in patients without underlying kidney disease (27). (3) Coagulation function abnormality was defined as a prolongation of prothrombin time (PT), activated partial thromboplastin time (APTT) or thrombin time (TT) by more than 25% from the baseline value, or a reduction of fibrinogen (FIB) by more than 25% from the baseline value (28). (4) Thrombocytopenia was defined as a reduction in platelet count (PLT) by more than 25% from the baseline value (28).

### 2.4 Statistical analysis

All statistical analyses were performed using SPSS 25.0 statistical software (IBM Corp., Armonk, NY, USA). Continuous variables conforming to normal distribution were tested by independent samples t-test and expressed as mean ± standard deviation. Continuous variables that did not conform to normal distribution were analyzed by non-parametric rank-sum test and expressed as median (interquartile range) [M (P25, P75)]. Categorical variables were presented as frequency and percentage (%), and inter-group comparisons were conducted using the chi-square (*χ*2) test or Fisher's exact test. Logistic regression analysis was performed to evaluate the risk factors associated with all-cause mortality. First, univariate logistic regression analysis was conducted. The associations between confounding factors and all-cause mortality, especially the impact of anti-CRKP treatment regimens, were preliminarily explored through the calculation of P values, odds ratios (ORs) and 95% confidence intervals (95% CIs). Subsequently, multivariable logistic regression analysis was performed. Confounding factors with *P* < 0.05 in the univariate analysis and the anti-CRKP treatment regimen were included in the multivariate model. A stepwise multivariable regression model was applied to explore factors associated with all-cause mortality, with a particular focus on the influence of anti-CRKP treatment regimens. The strength of the association between independent risk factors and all-cause mortality was assessed by calculating adjusted odds ratios and corresponding 95% CIs. In addition, multivariable logistic regression analyses were conducted for all primary efficacy and safety outcomes to assess the independent effect of treatment regimens. Age, comorbid diabetes mellitus, and concomitant other intravenous antibacterial agents were forced into all models as prespecified core confounders. For endpoints with limited events (28-day mortality and overall mortality), Firth's penalized logistic regression was used in place of conventional logistic regression to mitigate estimation bias, reduce overfitting risk, and improve the stability of effect estimates. Only prespecified core covariates were retained in all models to ensure parsimony. *P* < 0.05 was considered to indicate a statistically significant difference. No prospective sample size calculation was performed for this retrospective study, and statistical power was limited by the actual enrolled sample size.

## 3 Results

### 3.1 Comparison of basic clinical characteristics between the two groups

A total of 63 patients were enrolled in this study, with 33 assigned to the CAZ-AVI group (52.38%) and 30 to the TGC group (47.62%). Baseline characteristics were generally balanced between the two groups. No statistically significant differences were observed in demographic features (age, sex distribution, BMI), clinical baseline status (smoking history, alcohol use history, proportion of tracheotomy, proportion of multiple-site infection), comorbidities, study regimen treatment duration, or the proportion of concomitant nebulized antibacterial agents (all *P* > 0.05). However, a significant inter-group difference was detected in the proportion of concomitant other intravenous antibacterial agents (*χ*^2^ = 31.01, *P* < 0.001). Basic categorization of concomitant agents is presented in Table 1, and full detailed information including specific drug classes, daily dosages, duration of combination therapy, and available susceptibility data is provided in Supplementary Table S1.

**Table 1:** Comparison of baseline clinical characteristics between patients in the two treatment groups.

Variables	TGC group (*n* = 30)	CAZ-AVI group (*n* = 33)	U/*χ*^2^	*P*
Male (%)	21 (70.00)	28 (84.85)	2.005	0.157
Age (year)	71.87 ± 11.62	67.97 ± 16.36	−1.08	0.284
BMI (kg/m^2^)	22.45 ± 3.61	20.81 ± 2.91	−1.99	0.051
Smoking history (%)	7 (23.33)	9 (27.27)	0.129	0.720
Alcohol use history (%)	1 (3.33)	4 (12.12)	0.676	0.411
Tracheotomy (%)	13 (43.33)	14 (42.42)	0.005	0.942
Multiple-site infection (%)	6 (20.00)	10 (30.30)	0.880	0.348
Bloodstream	4 (13.33)	3 (9.09)	0.018	0.894
Central nervous system	1 (3.33)	2 (6.06)	0.000	1.000
Intra-abdominal	1 (3.33)	0 (0.00)		0.476
Urinary tract	0 (0.00)	4 (12.12)		0.115
Wound	0 (0.00)	1 (3.03)		1.000
Comorbidities (%)				
Coronary Artery Disease	7 (23.33)	4 (12.12)	0.703	0.402
Hypertension	17 (56.67)	16 (48.48)	0.422	0.516
Diabetes Mellitus	10 (33.33)	10 (30.30)	0.067	0.796
Hypoalbuminemia	20 (66.67)	24 (72.73)	0.274	0.601
Anemia	13 (43.33)	18 (54.55)	0.790	0.374
Electrolyte Disorders	8 (26.67)	8 (24.24)	0.049	0.825
Malignancy	4 (13.33)	2 (6.06)	0.305	0.581
Ischemic Stroke	13 (43.33)	13 (39.39)	0.101	0.751
Thromboembolism	10 (40.00)	13 (39.39)	0.002	0.961
Study regimen treatment duration (d)	8.53 ± 4.25	8.79 ± 3.62	0.26	0.798
Concomitant nebulized antibacterial agents (%)	6 (20.00)	4 (12.90)	0.260	0.610
Concomitant other intravenous antibacterial agents (%)	21 (70.00)	1 (3.03)	31.01	<.001
Carbapenems	14	0	–	–
Piperacillin	1	0	–	–
Cefoperazone	3	0	–	–
Third-generation cephalosporins	2	0	–	–
Polymyxin B	1	0	–	–
Levofloxacin	0	1	–	–

### 3.2 Comparison of therapeutic efficacy

**Figure 2:**
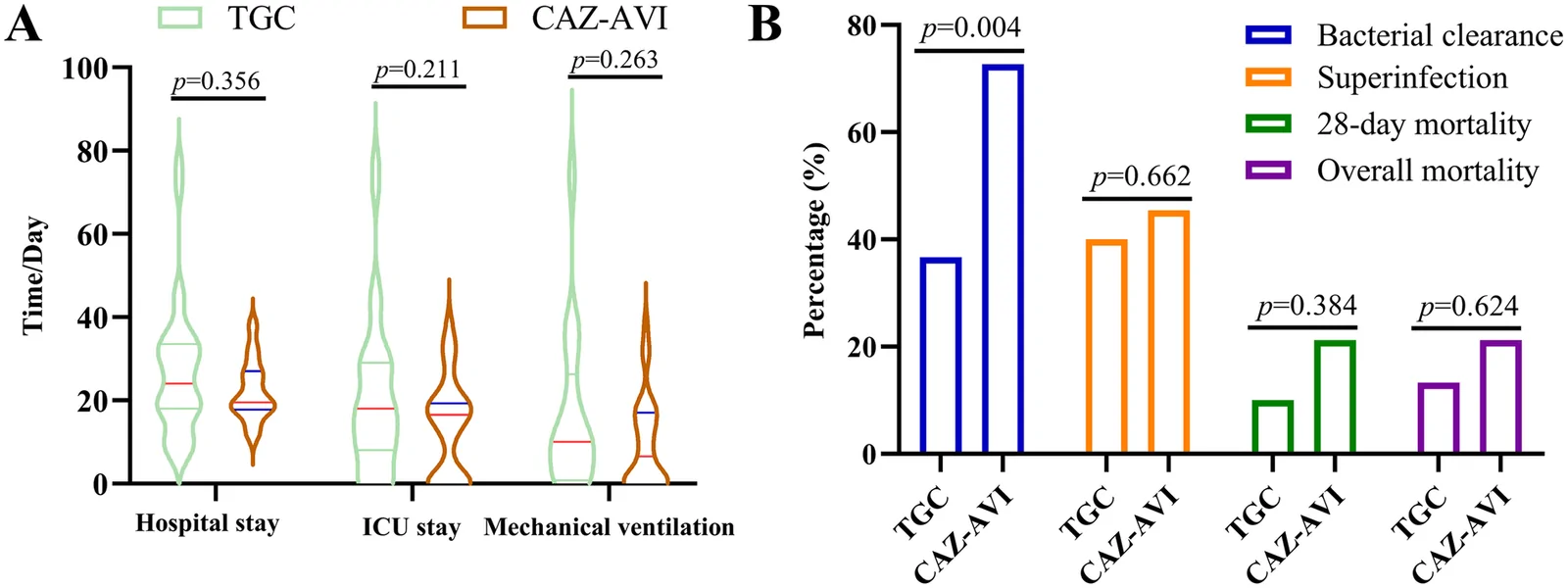
Comparison of therapeutic efficacy between TGC and CAZ-AVI groups. **(A)** Comparison of hospital stay, ICU stay and mechanical ventilation duration between the two groups. **(B)** Comparison of bacterial clearance, superinfection, 28-day mortality and overall mortality between the two groups.

No statistically significant differences were observed between the two groups in hospitalization-related indicators or clinical survival outcomes. No statistically significant between-group difference was observed in median length of hospital stay, ICU stay, or mechanical ventilation duration (all *P* > 0.05). Similarly, no statistically significant differences were detected in clinical response rate, superinfection rate, 28-day mortality, or overall in-hospital mortality (all *P* > 0.05). In contrast, the bacterial clearance rate was significantly higher in the CAZ-AVI group than in the TGC group (72.73% vs. 36.67%, *P* = 0.004). Detailed data are presented in Table 2 and Figure 2. To evaluate the confounding impact of concomitant antibacterial therapy, we performed a sensitivity analysis with multivariable adjustment for age, comorbid diabetes mellitus, and concomitant other intravenous antibacterial agents. In the sensitivity analysis, the direction of effect remained consistent with the crude analysis, with CAZ-AVI-based regimens showing a trend toward better efficacy across all endpoints; however, no statistically significant between-group differences were observed for any efficacy endpoint (all *P* > 0.05), partly due to reduced statistical precision caused by strong collinearity between treatment assignment and concomitant therapy. Full adjusted effect estimates are provided in Supplementary Table S2.

**Table 2:** Comparison of therapeutic efficacy between the two groups.

Variable	TGC group (*n* = 30)	CAZ-AVI group (*n* = 33)	Z/*χ*^2^	*P*
Length of hospital stay	19.50 (18.00, 26.75)	24.00 (18.00, 33.00)	−0.92	0.356
Length of ICU stay	16.50 (0, 19.00)	18.00 (8.00, 28.00)	−1.25	0.211
Duration of mechanical ventilation	6.50 (0, 16.75)	10.00 (0, 21.00)	−1.12	0.263
Clinical response (%)	12 (40.00)	20 (60.61)	2.670	0.102
Bacterial clearance (%)	11 (36.67)	24 (72.73)	8.276	0.004
Superinfection (%)	12 (40.00)	15 (45.45)	0.191	0.662
28-day mortality (%)	3 (10.00)	7 (21.21)	0.759	0.384
Overall mortality (%)	4 (13.33)	7 (21.21)	0.241	0.624

Z: Mann–Whitney test, *χ*^2^: Chi-square test. Among patients with bacterial clearance, 5 in the TGC group and 18 in the CAZ-AVI group had documented eradication (confirmed by negative follow-up culture); the remainder were classified as presumed eradication.

### 3.3 Comparison of treatment-emergent laboratory abnormalities

The incidences of hepatic and renal function abnormalities were low in both groups, with no statistically significant between-group difference detected (9.09% vs. 3.33% for hepatic function abnormality, *χ*^2^ = 0.18, *P* = 0.675; 6.06% vs. 3.33% for renal function abnormality, *χ*^2^ = 0.00, *P* = 1.000). However, CAZ-AVI-based regimens were associated with significantly lower incidences of both coagulation abnormalities and thrombocytopenia compared with TGC-based regimens (27.27% vs. 70.00%, *χ*^2^ = 11.50, *P* < 0.001; 15.15% vs. 43.33%, *χ*^2^ = 6.12, *P* = 0.013, respectively). Detailed data are presented in Table 3. After adjustment for the same confounders, the CAZ-AVI-based regimen remained independently associated with a significantly lower risk of coagulation abnormalities (adjusted OR = 0.02, 95% CI: 0.001–0.39, *P* = 0.010), while the between-group difference in thrombocytopenia showed a borderline nonsignificant trend (adjusted OR = 0.24, 95% CI: 0.05–1.11, *P* = 0.068). Full adjusted results are shown in Supplementary Table S2.

**Table 3:** Comparison of treatment-emergent laboratory abnormalities between the two groups.

Treatment-emergent laboratory abnormalities (%)	TGC group (*n* = 30)	CAZ-AVI group (*n* = 33)	*χ* ^2^	*P*
Abnormal hepatic function	1 (3.33)	3 (9.09)	0.175	0.675
Abnormal renal function	1 (3.33)	2 (6.06)	0.000	1.000
Abnormal coagulation function	21 (70.00)	9 (27.27)	11.501	<.001
Thrombocytopenia	13 (43.33)	5 (15.15)	6.115	0.013

### 3.4 Univariate and multivariate analysis of all-cause mortality

Univariate and multivariable logistic regression analyses were performed to explore factors associated with all-cause mortality in patients with CRKP pulmonary infection. Univariate analysis identified age, hypertension, and diabetes mellitus as variables significantly associated with mortality (all *P* < 0.05), which were subsequently included in the multivariate model. Firth penalized multivariable logistic regression showed that advanced age (adjusted OR = 1.12, 95% CI 1.03–1.22, *P* = 0.011) and comorbid diabetes mellitus (adjusted OR = 6.24, 95% CI 1.23–31.57, *P* = 0.027) were associated with increased all-cause mortality in this exploratory analysis. Given the limited number of mortality events and small overall sample size, these associations should be regarded as preliminary and hypothesis-generating, and interpreted with caution. Hypertension, although statistically significant in univariate analysis, lost its prognostic independence after adjustment for confounders (OR = 3.05, 95% CI 0.37–24.80, *P* = 0.298). Additionally, the choice of treatment regimen showed no significant impact on all-cause mortality between the two groups (*P* = 0.335). Detailed data are presented in Table 4.

**Table 4:** Univariate and multivariable analysis of all-cause mortality.

Variable	Univariate analysis				Multivariable analysis	
	Death (*n* = 11)	Survival (*n* = 52)	*P*	OR (95%Cl)	*P*	OR (95%Cl)
Group			0.414	1.75 (0.46∼6.71)	0.360	0.48 (0.10∼2.31)
TGC group (%)	4 (36.36)	26 (50.00)				
CAZ-AVI group (%)	7 (63.64)	26 (50.00)				
Male (%)	8 (72.73)	41 (78.85)	0.658	1.40 (0.32∼6.17)		
Age (year)	81.45 ± 7.62	67.37 ± 14.23	0.005	1.14 (1.04∼1.24)	0.011	1.12 (1.03∼1.22)
BMI (kg/m^2^)	20.36 ± 2.74	21.85 ± 3.42	0.182	0.85 (0.67∼1.08)		
Study regimen treatment duration (d)	8.64 ± 3.88	8.67 ± 3.94	0.977	1.00 (0.84∼1.18)		
Smoking history	4 (36.36)	12 (23.08)	0.363	1.90 (0.48∼7.63)		
Tracheotomy	3 (27.27)	24 (46.15)	0.259	0.44 (0.10∼1.84)		
Multiple-site infection	3 (27.27)	13 (25.00)	0.875	1.13 (0.26∼4.88)		
Bloodstream	2 (18.18)	5 (9.62)	0.419	2.09 (0.35∼12.49)		
CNS	1 (9.09)	2 (3.85)	0.472	2.50 (0.21∼30.29)		
Comorbidities						
Thromboembolism	6 (54.55)	19 (36.54)	0.273	2.08 (0.56∼7.76)		
Coronary artery disease	2 (18.18)	9 (17.31)	0.945	1.06 (0.20∼5.77)		
Hypertension	9 (81.82)	24 (46.15)	0.046	5.25 (1.03∼26.70)	0.305	2.49 (0.44∼14.21)
Diabetes mellitus	8 (72.73)	12 (23.08)	0.004	8.89 (2.03∼38.87)	0.027	6.24 (1.23∼31.57)
Hypoalbuminemia	8 (72.73)	36 (69.23)	0.819	1.19 (0.28∼5.06)		
Anemia	6 (54.55)	25 (48.08)	0.697	1.30 (0.35∼4.78)		
Electrolyte disorders	3 (27.27)	13 (25.00)	0.875	1.13 (0.26∼4.88)		
Tumor	2 (18.18)	4 (7.69)	0.296	2.67 (0.42∼16.80)		
Ischemic stroke	7 (63.64)	19 (36.54)	0.107	3.04 (0.79∼11.75)		
Concomitant nebulized antibacterial agents	1 (9.09)	9 (17.31)	0.479	0.46 (0.05∼4.02)		
Concomitant other-intravenous antibacterial agents	2 (18.18)	20 (38.46)	0.214	0.36 (0.07∼1.82)	0.262	0.42 (0.09∼1.93)

Adjusted odds ratios and 95% confidence intervals were derived from Firth's penalized logistic regression to account for the limited number of mortality events.

## 4 Discussion

**Figure 3:**
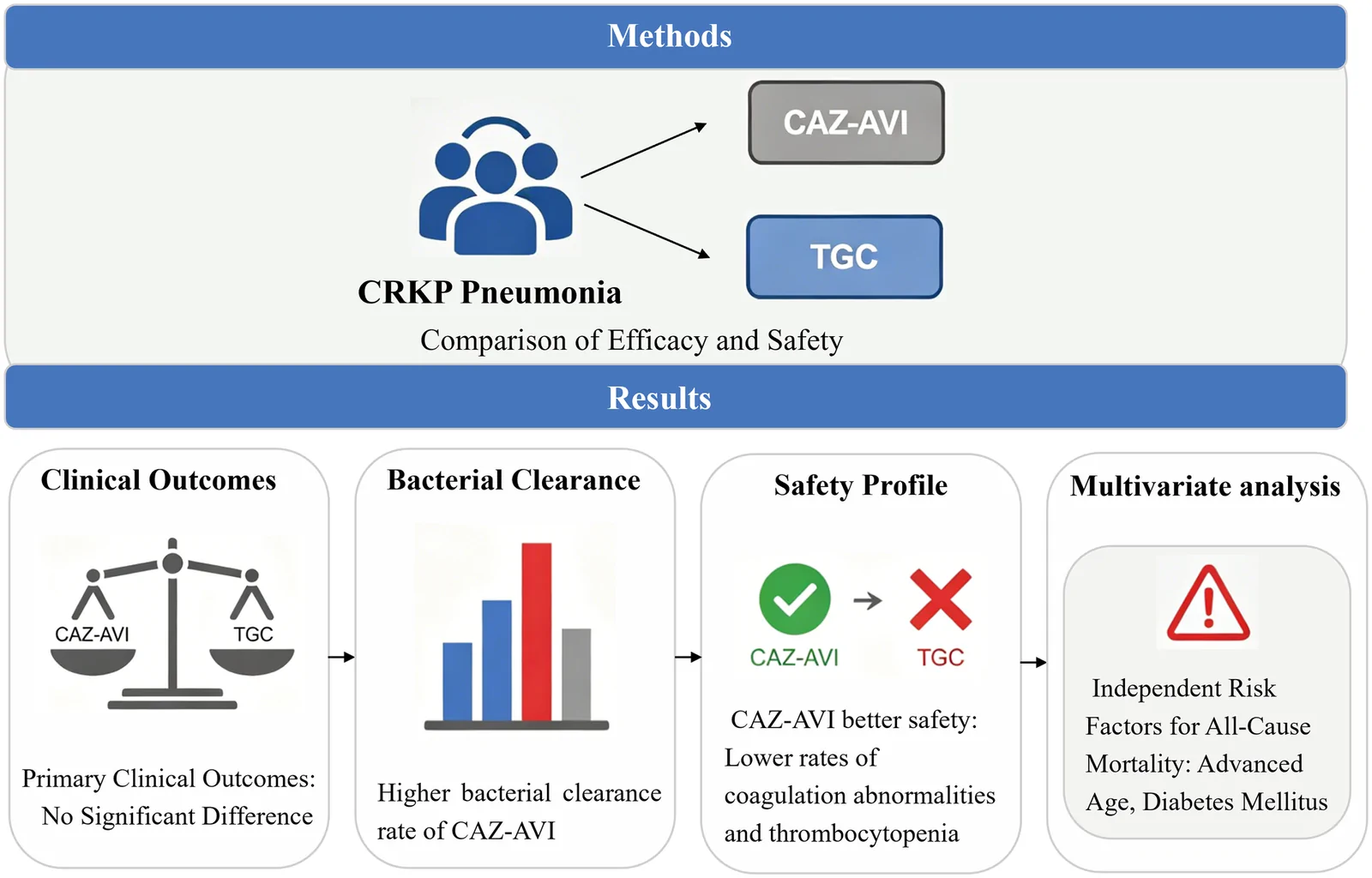
Graphical abstract: efficacy, safety and prognostic risk factors of ceftazidime-avibactam versus tigecycline for carbapenem-resistant *Klebsiella pneumoniae* pneumonia.

This retrospective study analyzed clinical data from 63 patients with pulmonary infection caused by CRKP, and systematically compared the clinical efficacy, microbiological eradication rate, safety profile, and prognostic outcomes between CAZ-AVI and TGC regimens. The results showed that, compared with TGC-based regimens, CAZ-AVI-based treatment was associated with a significantly higher pathogen eradication rate and a more favorable safety profile regarding coagulation function and platelet count, while no statistically significant differences were observed in clinical response, hospital stay, or all-cause mortality between the two groups. In addition, multivariable analysis detected exploratory associations of advanced age and diabetes mellitus with increased all-cause mortality in these patients (Figure 3). Although the overall superiority of CAZ-AVI over TGC for CRKP infections has been established in prior literature, this study provides targeted incremental evidence with notable clinical value. First, few existing studies focused exclusively on CRKP pneumonia, our study offers dedicated real-world data for this prevalent clinical subtype in intensive care settings in Northwest China. Second, we observed a lower incidence of coagulation abnormalities and thrombocytopenia associated with CAZ-AVI, supplementing exploratory safety data for clinical decision-making in critically ill patients with high bleeding risk. Third, we detected exploratory associations of advanced age and comorbid diabetes mellitus with all-cause mortality, providing preliminary prognostic clues rarely reported in previous comparative studies of the two regimens to inform future hypothesis-driven research. The findings of the present study are consistent with the core conclusions of several similar domestic and international studies (16, 19, 20), and the supplemented regional real-world data can provide a localized evidence-based reference for the individualized selection of antimicrobial regimens for CRKP pulmonary infection in clinical practice.

Regarding microbiological efficacy, the present study found that the bacterial clearance rate in the CAZ-AVI group was significantly higher than that in the TGC group; this finding is highly consistent with the results reported by Shi et al. in a study of critically ill patients with CRKP pneumonia in the ICU (16), which demonstrated a microbiological cure rate of 74.4% in the CAZ-AVI group, again significantly higher than the 33.9% in the TGC group (*P* < 0.001). The marked difference in microbiological eradication capacity between the two groups can be reasonably explained by their antibacterial mechanisms and pharmacokinetic/pharmacodynamic (PK/PD) profiles. Mechanistically, CAZ-AVI is a typical time-dependent bactericidal agent with potent activity against the endemic KPC-2-producing ST11 clone of CRKP in China (11, 29–31), whereas TGC exerts its effect solely by inhibiting bacterial protein synthesis, which confers an inherent limitation in bactericidal efficacy for critically ill patients with high bacterial load in pulmonary infection and constitutes one of the main reasons for its insufficient pathogen eradication capacity (32–34). In terms of PK/PD characteristics, CAZ-AVI achieves concentrations in epithelial lining fluid (ELF) of 30%–50% of concurrent plasma levels, which stably covers the minimum inhibitory concentration (MIC) for most CRKP isolates under standard dosing and efficiently attains the key pharmacodynamic target of %fT >MIC (11, 35, 36); in contrast, despite a favorable tissue penetration ratio into ELF, TGC yields substantially low systemic exposure in critically ill patients at conventional doses, and the PK/PD target attainment rate is less than 75% with standard dosing when the MIC of the isolate is ≥1 mg/L, making it difficult to maintain sustained and effective bactericidal concentrations at the pulmonary infection site and ultimately resulting in failed pathogen eradication (16, 17, 37, 38).

In terms of clinical response, the CAZ-AVI group demonstrated a clear trend of clinical benefit compared with the TGC group (60.61% vs. 40.00%), albeit without statistical significance. This result shows a certain degree of inconsistency with several prior studies documenting a significantly higher clinical cure rate for CAZ-AVI-based regimens (16, 19, 21). In-depth analysis of the underlying reasons revealed that this was first closely related to the baseline characteristics of the present study: the proportion of concomitant use of other intravenous antibacterial agents in the TGC group was as high as 70.00%, which was significantly higher than 3.03% in the CAZ-AVI group (*P* < 0.001), and current evidence-based data have clearly confirmed that TGC monotherapy is associated with high clinical failure rate and mortality in the treatment of CRKP pneumonia (38), thus combination with agents such as carbapenems and polymyxins is often applied in clinical practice to generate synergistic antibacterial effects to compensate for the insufficient efficacy of TGC monotherapy, and the high proportion of combination therapy in the TGC group in this study most likely narrowed the difference in clinical response rate between the two groups. The attenuation of statistical significance in the adjusted sensitivity analysis is attributable to two factors: first, adjustment for concomitant therapy partially corrected the baseline imbalance in disease severity, as combination regimens were preferentially used in more severe cases; second, strong collinearity between treatment group and concomitant agent use markedly reduced the precision of effect estimates and inflated the confidence intervals. Nevertheless, the consistent direction of effect across both crude and adjusted analyses supports the overall trend of superior microbiological activity with CAZ-AVI-based regimens. Secondly, only 63 patients were enrolled in this study, and the limited sample size may lead to insufficient statistical power, failing to detect the minor difference in clinical response rate between the two groups. Furthermore, the improvement of clinical symptoms in patients with CRKP pulmonary infection is the result of the combined action of multiple factors including pathogen eradication, host immune status, organ function, and supportive care (4, 39); all patients enrolled in this study were complicated with multiple underlying diseases such as hypertension, diabetes mellitus, and hypoalbuminemia, and the impact of host factors on clinical outcomes may have masked the efficacy difference of antibacterial agents themselves to a certain extent. In addition, there was no significant difference in the incidence of superinfection between the two groups, suggesting that the two treatment regimens exert similar disruptive effects on the normal intestinal flora of patients, and vigilance against the occurrence of superinfection is required regardless of which regimen is selected in clinical practice.

With regard to the safety profile, the results of this study showed that the incidences of abnormal hepatic function and abnormal renal function in both groups were at low levels, with no statistically significant difference between the two groups, demonstrating that both regimens have favorable hepatic and renal tolerability, which is consistent with the safety conclusions of existing domestic and international studies (16, 40, 41). The most critical safety finding of this study was that the incidence of abnormal coagulation function and thrombocytopenia in the TGC group reached 70.00% and 43.33%, respectively, which were significantly higher than the 27.27% and 15.15% observed in the CAZ-AVI group (all *P* < 0.05). After multivariable adjustment for baseline confounders, CAZ-AVI-based regimens remained independently associated with a significantly lower risk of coagulation abnormalities (adjusted OR = 0.02, *P* = 0.010), while thrombocytopenia showed a borderline nonsignificant trend toward reduced risk (adjusted OR = 0.24, *P* = 0.068). These findings indicate that the observed difference in coagulation safety cannot be attributed to baseline imbalances in age or diabetes status, and is consistent with prior evidence of an increased risk of coagulation abnormalities associated with tigecycline use. Previous studies have confirmed that tigecycline can induce coagulopathy through multiple mechanisms, including inhibiting the synthesis of hepatic coagulation factors, damaging vascular endothelial cells, and activating the fibrinolytic system; this adverse reaction exhibits a clear dose-dependent pattern, with a significantly elevated incidence in patients with severe infection, advanced age, and concomitant hepatic insufficiency (3, 20, 29). For patients with CRKP pulmonary infection, especially critically ill individuals with sepsis and multiple organ dysfunction, coagulation disorders can markedly increase the risk of bleeding events and even trigger disseminated intravascular coagulation (DIC), which directly endangers patients’ life safety; therefore, coagulation function and platelet count must be routinely monitored during the clinical application of TGC, with timely adjustment of the treatment regimen (42, 43). In contrast, the adverse reaction profile of CAZ-AVI is similar to that of traditional third-generation cephalosporins, with no confirmed adverse effects on the coagulation system, and this study is consistent with prior evidence of a lower risk of coagulation abnormalities with CAZ-AVI use; for patients with CRKP pulmonary infection complicated with coagulopathy, high bleeding risk, or in the perioperative period, CAZ-AVI-based regimens may have more favorable clinical applicability.

Regarding prognostic risk factors, Firth penalized multivariable analysis detected exploratory associations between advanced age, comorbid diabetes mellitus and increased all-cause mortality in patients with CRKP pulmonary infection, whereas the choice of treatment regimen exerted no significant impact on survival outcomes. This finding is consistent with multiple prior studies (3, 20, 39, 44), indicating that underlying host status, rather than the selection of antimicrobial regimen, is the core determinant of survival prognosis in CRKP pulmonary infection. Mechanistically, elderly patients generally have impaired immune function and progressive decline in multi-organ function, with significantly reduced compensatory and clearance capacity against infection, and the risk of death increases by 12% for each one-year increase in age, which is consistent with the conclusion from existing studies that advanced age is an independent risk factor for death in patients with CRE infection (39, 44). In contrast, patients with concomitant diabetes mellitus had a nearly 6-fold higher risk of death, and the core reason for this is that hyperglycemia can significantly inhibit the phagocytic and chemotactic functions of neutrophils and impair the body's innate immune barrier, while diabetes-related microangiopathy and cardiorenal function impairment can further aggravate the progression of infection and increase the risk of septic shock and multiple organ failure. Regarding the finding of no significant effect of the two treatment regimens on mortality, it is fully consistent with the conclusion of no statistically significant difference in 28-day survival rate between the two groups in the study by Shi et al. (16), and the main reasons include three aspects: first, the limited sample size of this study, with only 11 death events recorded, results in insufficient statistical power to detect minor differences in mortality between the two groups; to mitigate estimation bias caused by rare events, we applied Firth's penalized logistic regression in the multivariable analysis, and the observed associations should still be interpreted as preliminary and hypothesis-generating; second, the high proportion of combination antimicrobial therapy in the TGC group compensated for the insufficient efficacy of its monotherapy and narrowed the survival difference between the two groups; third, death in patients with CRKP pulmonary infection is the result of the combined action of multiple factors including the severity of infection, septic shock, organ failure, and host immune status, the core role of antibacterial agents is to eradicate pathogens, while the underlying host status and organ function ultimately determine the survival outcome of patients.

Nevertheless, several limitations of the present study should be acknowledged. First, this was a single-center retrospective study with a small sample size, which inevitably harbored selection bias and confounding factors; in particular, the significant difference in the proportion of concomitant antimicrobial therapy between the two groups may have confounded the efficacy and prognosis analyses. Full details of all concomitant antibacterial regimens have been disclosed for full transparency. We acknowledge that residual unmeasured confounding cannot be fully excluded in this retrospective single-center study, and all findings should be interpreted as exploratory observational associations rather than confirmatory causal evidence. Additionally, the limited sample size results in insufficient statistical power to confirm therapeutic equivalence between the two regimens; nonsignificant between-group differences should not be interpreted as equivalent efficacy. Second, molecular typing of carbapenemases was not performed for all CRKP isolates in this study, precluding stratified analysis of the differences in efficacy of the two therapeutic regimens across strains with different carbapenemase genotypes. Considering the local epidemiological context of Northwest China, clinically prevalent CRKP strains predominantly harbor KPC-2, a class A serine carbapenemase, while metallo-β-lactamases and class D carbapenemases account for a low proportion (10, 12). Therefore, the reduced activity of CAZ-AVI against strains producing non-class A carbapenemases exerts a relatively limited impact on the core conclusions of this study. Nevertheless, this limitation notably restricts the external generalizability of the findings; accordingly, our conclusions should be interpreted with caution and extrapolated prudently to regions where non-KPC carbapenemase genotypes are dominant. Third, this study did not conduct detailed stratified analysis of the dosing regimens and treatment duration of the two agents, nor did it implement therapeutic drug monitoring, thus failing to evaluate the impact of PK/PD target attainment on treatment efficacy and safety. Finally, this study had a limited follow-up duration, and no long-term tracking was performed for the long-term prognosis of patients after hospital discharge, as well as the emergence and transmission of drug-resistant strains following treatment. Additionally, the composite bacterial clearance endpoint including presumed eradication may introduce a slight overestimation bias, and relevant results should be interpreted with caution.

## 5 Conclusion

The present observational study found that for CRKP-induced pulmonary infection, CAZ-AVI-based regimens were associated with a higher bacterial clearance rate and a lower incidence of treatment-emergent coagulation and platelet abnormalities compared with TGC-based regimens. In regions where KPC-type carbapenemases are the dominant epidemic genotype, CAZ-AVI-based regimens may represent a reasonable therapeutic option for CRKP pulmonary infection. No statistically significant differences in clinical response rates or survival outcomes were detected between the two regimens in this study, and TGC-based combination regimens remain a vital alternative when CAZ-AVI is unavailable, the isolate shows confirmed CAZ-AVI resistance, or the patient has CAZ-AVI intolerance. Meanwhile, close attention should be paid to elderly patients with CRKP pulmonary infection and concomitant diabetes mellitus in clinical practice, as this population has a significantly elevated risk of death; while optimizing anti-infective treatment, it is essential to strengthen blood glucose management, organ function support and immune regulation to maximize the improvement of patients’ prognosis. This study provides supplementary single-center real-world data on CRKP pulmonary infection treatment in China. The results are intended to offer clinical reference for individualized regimen optimization, rather than definitive confirmatory evidence. In the future, multi-center, large-sample prospective studies are warranted to further validate the findings of this study.

## Data Availability

The raw data supporting the conclusions of this article will be made available by the authors, without undue reservation.
