# Supplementary material for: Efficacy and safety of ceftazidime-avibactam versus tigecycline in the treatment of pneumonia caused by carbapenem-resistant Klebsiella pneumoniae
Source: Front Med (Lausanne). 2026 Aug 28;13:1879917. doi: 10.3389/fmed.2026.1879917 (PMC13561761; doi:10.3389/fmed.2026.1879917)
Supplement: Supplementary file 1 [file Table1.docx]

**TABLE S1. Detailed information of study regimens and concomitant intravenous antibacterial agents in the two treatment groups**.

| **Study regimen drug** | | | | **Concomitant antibacterial agents** | | | | |
| --- | --- | --- | --- | --- | --- | --- | --- | --- |
| **Group** | **Dose (g)** | **Frequency** | **Treatment duration (d)** | **Drug** | **MIC** | **Dose (g)** | **Frequency** | **Treatment duration (d)** |
| TGC | 0.05 | 2 | 3 | Meropenem | ＞8 | 1 | 3 | 3 |
| TGC | 0.05 | 2 | 5 | Meropenem | ＞8 | 1 | 3 | 5 |
| TGC | 0.05 | 2 | 10 | Piperacillin/Tazobactam | ＞64 | 4.5 | 2 | 8 |
| TGC | 0.05 | 2 | 9 |  |  |  |  |  |
| TGC | 0.05 | 2 | 7 |  |  |  |  |  |
| TGC | 0.05 | 2 | 8 | Ceftazidime | ＞32 | 2 | 2 | 8 |
| TGC | 0.05 | 2 | 9 |  |  |  |  |  |
| TGC | 0.05 | 2 | 6 |  |  |  |  |  |
| TGC | 0.05 | 2 | 9 | Cefoperazone/Sulbactam | ＞32 | 3 | 2 | 9 |
| TGC | 0.05 | 2 | 9 | Cefoperazone/Sulbactam | ＞32 | 3 | 2 | 10 |
| TGC | 0.05 | 2 | 24 |  |  |  |  |  |
| TGC | 0.05 | 2 | 4 |  |  |  |  |  |
| TGC | 0.05 | 2 | 4 |  |  |  |  |  |
| TGC | 0.05 | 2 | 6 |  |  |  |  |  |
| TGC | 0.05 | 2 | 8 | Meropenem | ＞8 | 1 | 3 | 5 |
| TGC | 0.05 | 2 | 10 | Meropenem | ＞8 | 1 | 3 | 10 |
| TGC | 0.05 | 2 | 10 | Meropenem | ＞8 | 1 | 3 | 10 |
| TGC | 0.05 | 2 | 5 | Meropenem | ＞8 | 1 | 3 | 5 |
| TGC | 0.05 | 2 | 7 | Colistin | intermediate | 500,000 units | 2 | 5 |
| TGC | 0.05 | 2 | 6 | Ceftazidime | ＞32 | 2 | 2 | 6 |
| TGC | 0.05 | 2 | 12 | Imipenem | ＞8 | 1 | 3 | 15 |
| TGC | 0.05 | 2 | 10 | Meropenem | ＞8 | 1 | 3 | 10 |
| TGC | 0.05 | 2 | 16 |  |  |  |  |  |
| TGC | 0.1 | 2 | 6 | Meropenem | ＞8 | 1 | 3 | 5 |
| TGC | 0.05 | 2 | 7 | Meropenem | ＞8 | 1 | 3 | 2 |
| TGC | 0.05 | 2 | 10 | Meropenem | ＞8 | 2 | 3 | 10 |
| TGC | 0.05 | 2 | 15 | Meropenem | ＞8 | 1 | 3 | 15 |
| TGC | 0.05 | 2 | 4 | Meropenem | ＞8 | 1 | 3 | 4 |
| TGC | 0.05 | 2 | 11 | Cefoperazone/Sulbactam | ＞32 | 3 | 2 | 11 |
| TGC | 0.1 | 2 | 6 |  |  |  |  |  |
| AVI | 2.5 | 3 | 18 |  |  |  |  |  |
| AVI | 2.5 | 3 | 9 |  |  |  |  |  |
| AVI | 2.5 | 3 | 12 | Levofloxacin | ＞8 | 0.5 | 1 | 10 |
| AVI | 2.5 | 3 | 6 |  |  |  |  |  |
| AVI | 2.5 | 3 | 13 |  |  |  |  |  |
| AVI | 2.5 | 3 | 8 |  |  |  |  |  |
| AVI | 2.5 | 3 | 7 |  |  |  |  |  |
| AVI | 2.5 | 3 | 5 |  |  |  |  |  |
| AVI | 2.5 | 3 | 4 |  |  |  |  |  |
| AVI | 2.5 | 3 | 6 |  |  |  |  |  |
| AVI | 2.5 | 3 | 7 |  |  |  |  |  |
| AVI | 2.5 | 3 | 5 |  |  |  |  |  |
| AVI | 2.5 | 3 | 11 |  |  |  |  |  |
| AVI | 2.5 | 3 | 11 |  |  |  |  |  |
| AVI | 2.5 | 3 | 7 |  |  |  |  |  |
| AVI | 2.5 | 3 | 4 |  |  |  |  |  |
| AVI | 2.5 | 3 | 11 |  |  |  |  |  |
| AVI | 2.5 | 3 | 6 |  |  |  |  |  |
| AVI | 2.5 | 3 | 9 |  |  |  |  |  |
| AVI | 2.5 | 3 | 12 |  |  |  |  |  |
| AVI | 2.5 | 3 | 15 | Minocycline | 16 | 0.1 | 2 | 10 |
| AVI | 2.5 | 3 | 7 |  |  |  |  |  |
| AVI | 2.5 | 3 | 7 |  |  |  |  |  |
| AVI | 2.5 | 3 | 6 |  |  |  |  |  |
| AVI | 2.5 | 3 | 18 |  |  |  |  |  |
| AVI | 2.5 | 3 | 9 |  |  |  |  |  |
| AVI | 2.5 | 3 | 10 |  |  |  |  |  |
| AVI | 2.5 | 3 | 4 |  |  |  |  |  |
| AVI | 2.5 | 3 | 10 |  |  |  |  |  |
| AVI | 2.5 | 3 | 10 |  |  |  |  |  |
| AVI | 2.5 | 2 | 9 |  |  |  |  |  |
| AVI | 2.5 | 3 | 6 |  |  |  |  |  |
| AVI | 2.5 | 2 | 8 |  |  |  |  |  |

**TABLE S2. Adjusted associations between treatment regimen and efficacy and safety outcomes (multivariable logistic regression analysis)**

| **Efficacy and safety outcomes** | **Independent variable** | **Adjusted *P*** | **Adjusted OR** | **95% CI** |
| --- | --- | --- | --- | --- |
| **Efficacy outcomes** |  |  |  |  |
| Clinical response | CAZ-AVI vs. TGC (ref: TGC) | 0.348 | 2.15 | 0.43~10.69 |
| Bacterial clearance | CAZ-AVI vs. TGC (ref: TGC) | 0.331 | 2.09 | 0.47~9.29 |
| Superinfection | CAZ-AVI vs. TGC (ref: TGC) | 0.710 | 1.31 | 0.32~5.42 |
| 28-day mortality | CAZ-AVI vs. TGC (ref: TGC) | 0.983 | 1.02 | 0.14~7.48 |
| Overall mortality | CAZ-AVI vs. TGC (ref: TGC) | 0.963 | 1.05 | 0.13~8.35 |
| **Safety outcomes** |  |  |  |  |
| Abnormal coagulation function | CAZ-AVI vs. TGC (ref: TGC) | 0.010 | 0.02 | 0.001–0.39 |
| Thrombocytopenia | CAZ-AVI vs. TGC (ref: TGC) | 0.068 | 0.24 | 0.05–1.11 |

All models were adjusted for age, comorbid diabetes mellitus, and concomitant other intravenous antibacterial agents, with the TGC group set as the reference. Firth’s penalized logistic regression was applied for 28‑day mortality and overall mortality to mitigate estimation bias caused by limited events; standard multivariable logistic regression was used for all other outcomes. Owing to strong collinearity between treatment assignment and concomitant therapy, the adjusted point estimates should be interpreted with caution, and the analysis is primarily intended as a sensitivity assessment of confounding impact.
